# Breast cancer relapses considering molecular biological characteristics

**DOI:** 10.25122/jml-2022-0189

**Published:** 2023-01

**Authors:** Ivan Ivanovich Smolanka, Oleksii Volodimirovich Movchan, Irina Yuriivna Bagmut, Michael Ivanovich Sheremet, Igor Leonidovich Kolisnyk, Irina Viktorivna Dosenko, Andriy Oleksandrovich Lyashcnko, Oksana Mykolaivna Ivankova, Anton Dmitrovich Loboda, Oleksandr Viktorovich Shidlovskyi

**Affiliations:** 1 National Cancer Institute, Ministry of Health, Kyiv, Ukraine; 2 Kharkiv Medical Academy of Postgraduate Education, Kharkiv, Ukraine; 3 Department of Surgery No.1, Bukovinian State Medical University, Chernivtsi, Ukraine; 4 Department of Surgery, Ivan Horhachevsky Ternopil National Medical University, Ternopil, Ukraine

**Keywords:** breast cancer, relapse, molecular-biological properties of tumors, risk factors

## Abstract

We aimed to analyze the frequency of breast cancer relapses and their relationship with molecular and biological tumor characteristics. We studied 6,136 breast cancer patients, including 146 with relapses (Group 1) and 455 without relapses (Group 2). We divided the patients based on age, menstrual function, disease stage, histology form and grade, and molecular-biological subtype. The 5-year relapse-free rate for Group 1 was longer for Lum A and TN subtypes (60% and 40%, respectively) but shorter for Lum B and HER-2/neu-amplified subtypes (38% and 31%, respectively). Disease stage, tumor histology, and grade did not significantly affect relapse frequency in these patients. Relapses were more common in premenopausal patients and the Lum B subtype.

## INTRODUCTION

Breast cancer (BC) is the leading cancer among women in Ukraine, with 14,720 new cases reported in 2020 and a mortality rate of 28.4 per 100,000 population, which is higher than the global average of 14.0 per 100,000 population [1]. In 2020, 2.3 million new cases of BC were reported worldwide, with 685,000 deaths. According to the World Health Organization (WHO), by the end of 2020, there were 7.8 million women with breast cancer diagnosed in the past 5 years, making it the most widespread cancer in the world [2]. A study of the dynamic models of breast cancer shows that it is growing rapidly. The research indicates that over 37% of breast cancer cases and over 30% of deaths occur among working-age women, resulting in significant losses to the life potential of the female population in Ukraine [3].

From many postmastectomy disorders, the most significant concerns are recurrence development (7.9%), distant metastasis (15.5%), and the development of inflammatory complications (34%), even during the first year after the start of treatment [4].

Local relapse (LR) is a tumor process that occurs in the tissue of the chest wall after performing a radical operation on the breast cancer in the square, the lower edge of the collarbone, the ribs arc, and the middle and lateral axillary plots as the original tumor [5]. Regional relapse is the emergence of metastasis in regional lymph nodes after surgical or combined treatment, regardless of lymph dissection [6].

After performing radical mastectomy (RME), local relapse occurs within three months to decades. In addition, if LR occurs within two years after mastectomy, the forecast of the disease is very unfavorable. The frequency of LR in patients with I-IIA stages was 5–10% of cases after five years of surgical treatment, and ten years after treatment reached 25–30% [7].

After organ-preserving operations (OPA) on breast cancer, the frequency of LR after 5 years of treatment amounted to 10–15% [8]. According to a multi-center study with a median observation period of 62 months, recurrences occurred in 469 patients. These included 237 cases (50.6%) in the anterior breast wall, 235 cases (50.1%) in the subclavicular lymph nodes, 93 cases (20.7%) in the axillary lymph nodes, and 51 cases (11.7%) in the internal breast lymph nodes. The rate of relapse within five years was 3.7% and 5.3%. The most common site of regional recurrence was the chest wall and supraclavicular/subclavicular/axillary lymph nodes [9].

A rare form of breast cancer known as invasive micropapillary carcinoma (IMPC) is frequently discussed because of its propensity for lymph node involvement and challenges with precise imaging assessment. Larger tumors, higher histological grades, and a noticeably higher proportion of lymph nodes positive for the illness are all prominent characteristics of micropapillary carcinomas. IMPCs are more likely than other NST carcinomas to be hormone- and HER-2-positive. IMPC can manifest on its own or more frequently as a component of mixed non-specific type (NST) cancer [10]. According to the most recent statistics, effective surgical treatment frequently necessitates expanded surgical margins and diligent preoperative axillary staging due to a higher prevalence of lymph node invasion and locoregional recurrence, even though these histological subtypes of breast cancer have identical survival rates to other histological subtypes of breast carcinoma [10]. Pure micropapillary carcinoma is a rare type of breast cancer, representing just 0.9–2% of all breast carcinomas, whereas micropapillary histological architecture can be seen in up to 2–8% of all breast cancers. Many traits make up this histological pattern, which is surprisingly both elusive and aggressive, and these characteristics make invasive micropapillary carcinoma an emerging oncological and surgical problem [11].

However, according to other studies, the presence of an intraductal component increases the risk of LR by 15% within 5 years, particularly in cancer tumors larger than 5 cm and with low grade [12]. The mucinous and medullary forms of breast cancer have a lower risk of relapse at 3.7% and 2.3%, respectively, while the tubular form has a higher risk at 32.7%. Risk factors for relapse include lymph node damage and lack of adjuvant treatment, which in such forms is not carried out [13].

If the "purity of edges of resection" is less than 1 cm, the risk of relapse increases four times [14] unless the patient received radiation therapy in the postoperative period [15].

Younger patients with aggressive forms of breast cancer, such as large tumors, lymph node involvement, negative steroid hormone status, low tumor differentiation, and high expression of HER-2/neu, are at a higher risk of relapse and distant metastases [16].

Hormones-independent forms of breast cancer, such as those containing estrogen receptors (ER) and progesterone (PR), have a more favorable course. The effect of endocrine treatment is 50% for estrogen-positive (ER+) tumors, and this effect increases up to 60–70% for ER+ and PR+ tumors. In the absence of positive receptors on endocrine therapy, only 5–10% of patients respond to endocrine therapy on average. However, 30% of patients respond to endocrine therapy even with unknown receptor status [17].

The risk of LR was studied in two randomized studies, PP (3739 patients with primary-operable BC received only surgical treatment, and 1688 also adjuvant hormone therapy) and the SPORE study (10444 patients receiving adjuvant hormone therapy). The risk of LR in BC patients with ER + and PR + was less than 53%, and ER + and PR ess than 25%, compared to the ER-negative and PR-negative. However, HER-2-positive tumors are aggressive and resistant to chemotherapy (in patients with IIA-B – III stadium), which leads to a decrease in 5-year survival rates and a three-fold increase in 10-year survival rates [18].

Triple-negative breast cancer (TNBC) ranges from 11% to 22% of all phenotypical variants of BC and is mainly found in patients under 40 years. TNBC refers to the aggressive forms of BC and is represented by low-grade protocol cancer [19, 20].

The carriers of BRCA1 and BRCA2 mutations have a high risk of developing breast cancer, with a 33–50% chance at 50 and a 55–85% chance at 70 years [21]. The frequency of these mutations in families with a history of breast cancer is 21%. The presence of these mutations also increases the likelihood of cancer relapse, with a 21.8% relapse rate compared to 12.1% in the general population. Most relapses in BRCA-associated breast cancer occur early after surgery [22]. BRCA-1 is associated with 80% of basal-cellular phenotypes. In addition, 60% of BRCA2-associated tumors express EGFR [23].

This study aimed to analyze the nature and frequency of breast cancer relapses, taking into account molecular and biological characteristics of the tumor, the stage of the disease, histological structure, differentiation of the tumor, menstrual status, and the development of local relapses.

## MATERIAL AND METHODS

From 2010 to 2020, 6,136 breast cancer patients underwent surgeries and were followed up, including 146 patients with relapse (first group) and 455 patients without relapse (second group). Patients were distributed according to age, menstrual function, disease stage, histology form and grade, and molecular-biological subtype. All patients underwent histological and immunohistochemical verification of the diagnosis using steroid hormone receptors, Her-2/neu, Ki-67, and VEGF.

The following antibodies were used for immunohistochemical determination of estrogen, progesterone receptors, HER2/neu, Ki-67, estrogen receptor (Monoclonal Rabbit Anti-Human, RTU, clone SP1, Dako), progesterone receptor (Monoclonal Rabbit Anti-Human, RTU, clone PgR 636, Dako), c-erB2 (Polyclonal Rabbit Anti-Human, 1:1000, Dako), Ki-67 (Monoclonal Mouse Anti-Human, RTU, clone MIB- 1, Dako). ABC-Kit (universal) from Novocastra was used as the visualization system. Pharmab DAB was used to detect the coloring of the reaction result.

They performed recognizable proof of erBb2 amplification using fluorescence in situ hybridization (FISH) and immunohistochemistry (IHC) for HER-2 overexpression. Advanced characterization of DNA was performed using digital droplet PCR (ddPCR) and low-coverage whole genome sequencing (lcWGS). DdPCR is a powerful and accurate method for determining the copy number (CN) of a specific DNA segment. LcWGS detects DNA amplification and deletions throughout the genome and amplicon structure (AS). By combining these methods, we were able to produce detailed amplicon CN and AS. Histograms were created using Microsoft Excel and Statistica version 7.

## RESULTS

In our study, most patients were aged between 40 to 59 years old and had varying menstrual status (Table 1). Therefore, we analyzed the menstrual status in the selected groups of patients (Table 2).

**Table 1:** Breast cancer distribution among patients depending on age.

Age of patients	First group n (%)	Second group n (%)
**30–39**	27 (18.5±3.2%)	42 (9.2±1.4%)
**40–49**	56 (38.3±4.0%)	123 (27.0±2.1%)
**50–59**	51 (34.9±3.9%)	262 (57.6±2.3%)
**60–69**	12 (8.2±2.3%)	28 (6.2±1.1%)
**All**	146 (100.00)	455 (100.00)

The average age of the patients studied ranged from 30 to 69 years (45±0.8), p<0.05.

**Table 2:** Breast cancer distribution among patients depending on the menstrual status.

The state of the menstrual function of patients	Pre-menopause n (%)	Post-menopause n (%)	All
**1^st^ group**	67 (45.9±4.1%)	79 (54.1±4.1%)	146
**2^nd^ group**	213 (46.8±2.3%)	242 (53.2±2.3%)	455

The distribution of patients was homogeneous among the first and second groups, P<0.05.

We observed no significant differences in the distribution of patients based on menstrual status indicators. Our analysis of patient menstrual status data revealed that local relapses occur 8.7% more frequently in postmenopausal women over the age of 49.

According to the histological structure, ductal carcinoma was the most common type in both groups. In the first group, ductal carcinoma was present in 89 (61.0±4.0%) patients, and in the second group, in 265 patients (58.2±2.3%). Lobular carcinoma was diagnosed in 25 patients (17.1±3.1%) in the first group and 112 (24.6±2.0%) in the second group (p<0.05). Mixed lobular-duct carcinoma was found in 18 patients in the first group (12.3±2.7%) and 52 (11.4±1.4%) in the second. Medullary carcinoma was present in 5 patients (3.4±1.5%) in the first group and 9 patients (2.1±0.7%) in the second group (P<0.05). Finally, mucosal carcinoma was present in 9 patients (6.2±2.0%) in the first group and 17 patients (3.7±0.9%) in the second group (Table 3).

**Table 3:** Breast cancer distribution among patients depending on the histological structure of the cancer.

Histological structure of the cancer	1^st^ group n (%)	2^nd^ group n (%)	P-value
**Infiltrative lobular**	25 (17.1±3.1%)	112 (24.6±2.0%)	P<0.05
**Infiltrative ductal**	89 (61.0±4.0%)	265 (58.2±2.3%)	P<0.05
**Mix lobular-ductal**	18 (12.3±2.7%)	52 (11.4±1.4%)	P<0.05
**Mucosal**	9 (6.2±2.0%)	17 (3.7±0.9%)	P<0.05
**Medullar**	5 (3.4±1.5%)	9 (2.1±0.7%)	P>0.05
**All**	146 (100.00)	455 (100.00)	-

The absolute majority of patients in both the first and second groups had IIA and IIB stages at the time of diagnosis (Table 4).

**Table 4:** Breast cancer stages.

Stage	1^st^ group n (%)	2^nd^ group n (%)	P-value
**I (T1N0M0)**	23 (15.7±3.0%)	151 (33.1±2.2%)	P<0.05
**II A (T1N1M0, T2N0M0)**	63 (43.2±4.1%)	196 (43.1±2.3%)	P<0.05
**II B (T2N1M0, T3N0M0)**	40 (27.4±3.7%)	81 (17.8±1.8%)	P<0.05
**III A (T2N2M0)**	18 (12.3±2.4%)	19 (4.2±0.9%)	P<0.05
**III B (T4N0M0, T4N1M0)**	2 (1.4±1.0%)	8 (1.8±0.6%)	P<0.05
**All**	146 (100.00)	455 (100.00)	-

The estimation of tumor histology' and grade does not significantly affect the relapse frequency in inflammatory breast cancer (IBC) patients (Table 5).

**Table 5:** Breast cancer distribution among patients depending on tumor grade.

Tumor grade	1^st^ group n (%)	2^nd^ group n (%)	P-value
**G I**	22 (15.1±3.0%)	115 (25.3±2.0%)	P<0.05
**G II**	96 (65.7±3.9%)	276 (60.6±2.3%)	P<0.05
**G III**	19 (13.0±2.8%)	43 (9.5±1.4%)	P<0.05
**G IV**	9 (6.2±2.0%)	21 (4.6±1.0%)	P<0.05
**All**	146 (100.00)	455 (100.00)	-

Luminal A subtype occurred in 26.7% of patients in the first group and 24.4% in the second group. The Luminal B subtype was present in 46.6% of patients in the first group and 57.8% in the second group.

The HER-2/neu subtype, with confirmed amplification (3+), was present in 7.5% of patients in the first group and 5.3% in the second group. The triple-negative subtype occurred in 19.2% of patients in the first group and 12.5% in the second group (Table 6).

**Table 6:** Tumor molecular-biological characteristics.

The molecular-biological subtype	1^st^ group n (%)	2^nd^ group n (%)	P-value
**Luminal A**	39 (26.7±3.7%)	111 (24.4±2.0%)	P<0.05
**Luminal B**	68 (46.6±4.1%)	263 (57.8±2.3%)	P<0.05
**Her 2 types (3+)**	11 (7.5±2.2%)	24 (5.3±1.0%)	P<0.05
**Triple-negative**	28 (19.2±3.3%)	57 (12.5±1.6%)	P<0.05
**All**	146 (100.00)	455 (100.00)	-

**Figure 1:**
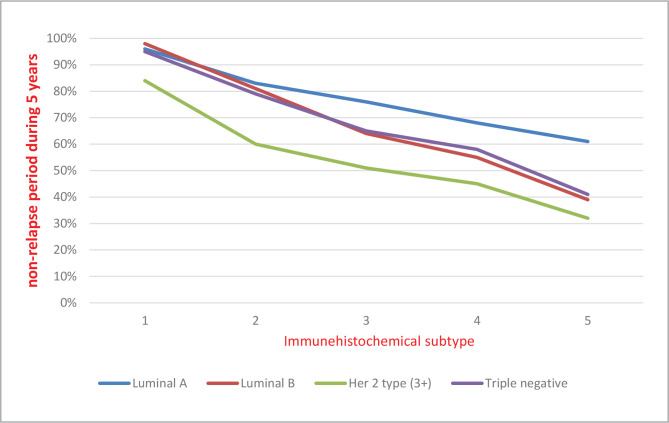
Distribution of non-relapse period in patients from Group 1 according to the molecular-biological subtypes of the tumor over 5 years.

**Figure 2:**
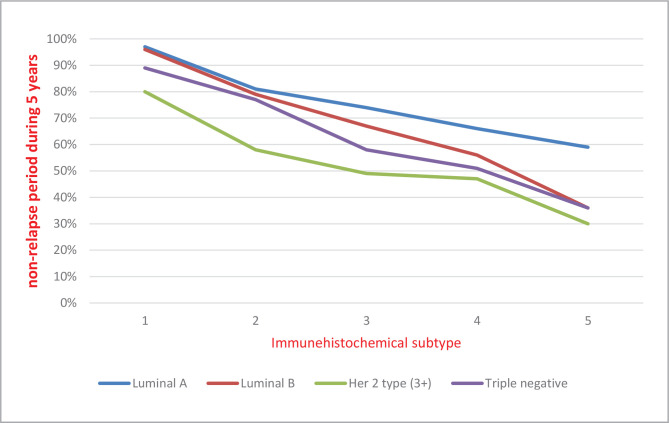
Distribution of non-relapse period in patients from Group 2 according to the molecular-biological subtypes of the tumor over 5 years.

Our data show that the 5-year non-relapse period is longer in patients from the first group with Luminal A and triple-negative subtypes (60% and 40%, respectively) Figure 1, compared to those with Luminal B and HER-2/neu subtypes with confirmed amplification (3+) (38% and 31%, respectively) Figure 2.

## DISCUSSION

According to the histological structure, ductal carcinoma was the most common type, consistent with the findings of previous research by Belhadj *et al*. [24], across all age categories but less frequently in older patients. This contrasts with Li Wang *et al*. [25], who found that molecular typing only accounted for 15% of predicting prognosis of breast cancer relapses compared to 60% for TNM staging and 25% for histological grading. Our findings support the idea that the initial grade of the tumor is a crucial factor in determining local relapse in breast cancer patients, as suggested by Johansson *et al*. [26]. Our analysis of patient menstrual status data revealed that local relapses occur 8.7% more frequently in postmenopausal women over the age of 49, corresponding with Orgéas [27].

## CONCLUSION

In conclusion, our study found that stage, tumor histology, and grade do not have a significant impact on relapse frequency in inflammatory breast cancer patients. The relapses occur more frequently in the Luminal B subtype, while the least relapses occur in the Luminal A subtype. Relapses are more common in premenopausal women.

## Personal thanks

We want to thank Professor Irina Yurievna Bagmut.
